# Mesh-associated pain syndrome: predictors for continence and prolapse mesh removal surgery in a single centre

**DOI:** 10.1186/s12905-024-03393-5

**Published:** 2024-11-01

**Authors:** Sohier Elneil, Gayathri Delanerolle, Yutian Zeng, Deng Chunli, Ashish Shetty, Jian Qing Shi

**Affiliations:** 1 University College London, London, UK; 2 University College London Hospitals NHS Foundation Trust, 235, Euston Road, London, NW1 2BU UK; 3 University of Birmingham, Birmingham, UK; 4 Southern University of Science and Technology, Shenzen, China; 5 Southern Health NHS Foundation Trust, Southampton, UK

**Keywords:** Continence mesh, Prolapse mesh, Comorbidity, Surgery

## Abstract

**Objective:**

Over the last two decades one of the main surgical treatment for stress urinary incontinence (SUI) and pelvic organ prolapse (POP) surgery was the insertion of non-absorbable mesh to restore continence and prolapse respectively. Over time complications arose including mesh-associated pain syndrome (MAPS), mesh exposure, mesh, erosion, chronic bladder/vaginal infections, and dyspareunia. Consequently, women chose surgical mesh removal to counter these problems. However, little is known about the demographics, medical co-morbidities, mesh types involved and the timing from mesh insertion to mesh removal. This retrospective study will look at which of these factors may be closely associated with mesh removal surgery.

**Design:**

Retrospective evaluation.

**Setting:**

Female Pelvic Medicine and Reconstructive Surgery (FPMRS) Clinic at University College London Hospitals NHS Foundation Trust.

**Population:**

All patients presenting to the FPMRS Clinic between June 2011 to December 2019, requesting mesh removal surgery with a history of MAPS and other mesh complications were included in this study.

**Methods:**

Patient demographics including age, ethnicity, obstetric history, and medical co-morbidities; type of mesh/anatomical route used; onset of symptoms; and time from insertion to removal were recorded.

**Main Outcome Measures:**

Determination of correlation coefficients between patient demographics, patient reported symptoms and mesh removal surgery.

**Results:**

Three hundred and forty-five women with a history of MAPS were included in the study. Women in the 40–60 year old cohort accounted for 54.4% of mesh removal surgery; 54.8% had a BMI under 30 and almost 90% were Caucasian. 96.5% had had children, with over 77% having had a vaginal delivery. 91.9% of patients reported other health conditions including 18.8% with a concomitant history of mental health problems and 15.4% with a history of heart disease. Over 80% of women undergoing mesh removal surgery had a continence mesh (49% retropubic and 32% obturator continence mesh) removed, whereas 20% had an abdominal prolapse and/or vaginal prolapse mesh removed. The average time from mesh insertion to mesh removal was seven years, with the prevalence of mesh removal surgery averaging 85% (range 50–100%) depending on the comorbidity determined.

**Conclusions:**

All women presented to the clinic with a history of MAPS and other comorbidities which may have influenced their decision to pursue mesh removal surgery. There were no specific predictors, other than chronic pain associated with mesh, determining which women underwent surgery, though those with continence mesh were more likely to do so.

## Introduction

Up until 2020, the surgical management of stress urinary incontinence (SUI) and pelvic organ prolapse (POP) was often associated with the use of biological and synthetic meshes, which are implanted as the sole intervention or in addition to other types of surgery. Biological meshes included native patient tissue, allografts and xenografts which were rarely used as they provided inconsistent tissue strength and lead to high POP and SUI recurrence rates [1, 2]. Thus, the vast majority of meshes used were synthetic and derived from polypropylene [2], and are classified as Amid Type 1 [3] absorbable synthetics, which had hitherto been used in hernia repairs. All such meshes, whether used in abdominal wall hernia repairs [4], POP or SUI were associated with chronic pelvic pain which in association with other symptoms such as vaginal, sexual, bladder, bowel and abdominal pain became known as mesh-associated pain syndrome (MAPS). The term mesh-related pain syndrome of MAPS was first used in the European Association of Urology Chronic Pain Guidelines of 2014. The importance of recognising the harm and/or injury associated with mesh or grafts was recognised in ICD-11 and validated/coded as PK96 (https://icd.who.int/browse11/l-m/en#/http%3 A%2 F%2Fid.who.int%2Ficd%2Fentity%2F69547070) in 2018. Other mesh complications include mesh erosion or extrusion [5, 6]. Procedures with the highest risks of erosion and pain, requiring reoperation procedures for POP [1, 7–10] were the vaginal mesh kits used for POP [11, 12].

Although female patients have been reporting problems following pelvic mesh surgery for a number of years, there has been a lack of robust evidence based-data to conclusively support the use or ban of these devices from clinical practice until recently. Past FDA approval for some of the pelvic meshes used poor quality evidence, many devices gaining approval through the use of ‘substantial equivalence’ data from previous research, and inconsistent post marketing surveillance [13].

In October 2008 and July 2011 the U.S Food and Drug Administration (FDA) issued a safety communication, warning about ‘not rare’ and ‘serious’ complications associated with surgical mesh devices used for SUI and POP repair. The incidence of reported complication rates between 2005–2007 was over 1000, rising to 2974 between 2008–2010. The FDA’s systematic review conducted between 1999–2011 concluded that ‘transvaginal POP repair with mesh does not improve symptomatic results or quality of life over traditional non-mesh repair’ and ‘ introduces risks not present in traditional non-mesh surgery for POP repair’ [10]. In 2019 the US FDA ordered all manufacturers of transvaginal surgical mesh to stop selling and distributing their products.

In 2005 the Cochrane review for NICE highlighted the lack of reliable evidence to support the use of sub urethral mesh operations in the management of SUI [14]. Although evidence for the approval of some transvaginal mesh products was weak [13], they remained a popular treatment for SUI and POP. As mesh-related problems continued to emerge, more women openly voiced their concerns about their continued use in SUI and POP. This was echoed by large groups of patients in the UK such as the 10,000 member Sling the Mesh Facebook support group and the 100,000 women in the US who are in an ongoing litigation process for mesh related complications. Since the 2005 report, Cochrane has updated their Cochrane review in 2015 and 2017 indicating the updated data shows a good safety profile. However, Cochrane indicates that longer-term outcome data is required to have a better understanding of mesh-related complications. Additionally, it is vital to understand that the Cochrane review included studies with a collective sample of 12, 113 with minimal demographic information that is vital to understand any possible correlations and underlying issues.

The widespread use of mesh in the management of incontinence has resulted in more patients being referred for management of their chronic pain symptoms, or MAPS. In the last decade [15], these complaints are more widely reported and the United Kingdom government agencies, the Medical Healthcare Regulatory Authority and the National Institute for Health and Clinical Excellence, have had to address them. This.

Rising awareness of mesh-related complications has caused large regulatory bodies and national institutions to issue cautions and warnings about the use of pelvic mesh products. This culminated in the Cumberlege Report *First Do No Harm*, published in July 2020 [16].

The slow and delayed withdrawal and suspension of transvaginal mesh in POP and SUI surgery, has been partly a result of poor evidence-based practice. To date there have been few, large scale population-based studies looking at the short- and long-term complications of transvaginal mesh in women with POP and SUI.

Studies looking at pelvic mesh complications to date have reported up to 30% rates of chronic pelvic pain or MAPS after transvaginal mesh surgery [17] due to a combination of pelvic floor spasm, pudendal or obturator nerve neuralgia, sub-clinical mesh infection [17] and chronic inflammation. However, pre-existing risk factors such as CPP prior to pelvic surgery [18], signs and symptoms of pudendal neuralgia [19] and the use of trocars for transvaginal tape insertions [20] can compound the situation. Barks et al. showed mid-urethral slings account for chronic pelvic pain and dyspareunia in 14% and 6% of patients respectively, whilst Barski et al. estimated transvaginal mesh in POP caused dyspareunia in 11% of patients [21].

The majority of mesh-related complications require a series of surgical procedures to remove the device and repair the POP or SUI that often recur [7, 9, 17, 19, 22], and/or chronic pain management. The former is by no means a panacea for resolution of all their symptoms, as 20–29% of those undergoing mesh removal surgery for chronic pain or dyspareunia do not get full relief for symptoms [18, 22, 23]. Many continue to experience chronic pelvic pain, pelvic floor dysfunction and psychological distress; often requiring long-term care and support [23, 24]. No such study has been completed in the UK.

This is the first cohort specific audit review completed to better evaluate patient demographics, patient reported symptoms, and comorbidities in women seeking mesh removal surgery in the UK as a consequence of mesh-related pain syndrome.

## Methodology

### Dataset

The individual patient dataset was gathered as part of an audit to obtain insights with mesh complications at University College London Hospitals NHS Foundation Trust. The cohort is consistent of patients who have had surgical mesh removal due to mesh-associated pain syndrome (with a view to reducing the mesh load significantly) along with details of their demographics, comorbidities, and patient reported symptoms gathered as part of a hospital visit.

### Data processing

During the data processing phase, we identified a significant level of missing data and inconsistencies associated with reporting of the data, although this is a common problem within electronic healthcare record systems. Following the processing, a table was curated with using abbreviations as demonstrated in Table 1.

**Table 1:** Explanation of abbreviations

Terminology	Definition used for the statistical analysis	Abbreviation used
Age at first surgery (removal)	Patient’s age at the first mesh removal surgery	AF Removal
BMI	Body Mass Index	BMI
Para	Number of deliveries (vaginal + caesarean)	Para
Caesarean Section		CS
3/4-degree tear	Obstetric anal sphincter injury (OASI)	OASI
FD/VO	Forceps Deliver or Ventouse Delivery	FD/VO
Ethnicity	1: Caucasian 2: Asian 3: African	Ethnicity
Medical History 1	A full-scale medical history for potential complications:	MH_1
Medical History 2	1fybromialgia 2diabetes 3 Other Endocrine 4 Autoimmune 5 Psychiatric 6 IBS 7 Cardiovascular 8 COPD/Asthma 9 Neuro 10 Disc Prolapse	MH_2
Autoimmune disorder	1 Spondylitis 2 Rheumatic Arthritis 3 EDS 4 Joint Hypermobility 5 Positive Auto-AB 6 Osteoarthrosis 7 Psoriasis/Psoriasis Arthritis	AD
Other medical	Other co-morbidity not defined specifically	OM
Year first mesh insertion	Specific year for the first mesh insertion surgery	YFMI
N years beginning of symptoms	Number of years for beginning of certain symptoms	N_BS
N years insertion-removal	Time interval between mesh insertion and removal surgery	N_IR
TVT	Tension free vaginal tape	TVT
TVT-O	Trans-obturator midurethral sling from inside to outside	TVT-O
TOT	Transobturator tape	TOT
Continence mesh count	Number of continence meshes, sum of TVT, TVT-O and TOT	CM_count
SCP	Sacrocolpopexy	SCP
SHP	Sacrohysteropexy	SHP
VMR	Ventral rectopexy mesh	VMR
Anterior wall mesh	Number of mesh put in Anterior wall	AWM
Posterior wall mesh	Number of mesh put in Posterior wall	PWM
Prolapse mesh count	Number of Prolapse meshes, sum of SCP, SHP, VMR, Anterior and Posterior wall mesh	PM_count
Total mesh count	Total number of meshes inserted, sum of Continence meshes and Prolapse meshes	TM_count

### Statistical methods

Logistic regression method applied for the purpose of this study is as follows;

$$\:\text{P}({y}_{i}=1|{x}_{i1},{x}_{i2},…,{x}_{ip})\:=\frac{1}{1+{e}^{-({\beta\:}_{0}+{\beta\:}_{1}*{x}_{i1}+{\beta\:}_{2}*{x}_{i2}+\dots\:+{\beta\:}_{p}*{x}_{ip})}}\:,\:i=\text{1,2},\dots\:,n$$

#### X

explanatory variables.

$\:{x}_{1},{x}_{2},\dots\:,{x}_{p}$ are vectors with length n, where

$$\:{x}_{1}=\left[\begin{array}{c}{x}_{11}\\\:\dots\:\\\:{x}_{n1}\end{array}\right],\:{x}_{2}=\left[\begin{array}{c}{x}_{12}\\\:\dots\:\\\:{x}_{n2}\end{array}\right],{\dots\:,x}_{p}=\left[\begin{array}{c}{x}_{1p}\\\:\dots\:\\\:{x}_{np}\end{array}\right]$$

#### Y

response variable.

$$\:{y}_{i}=\left\{\begin{array}{cc}0,&\:no\:removal\\\:1,&\:removal\end{array}\right.$$

In addition, descriptive statistics was used to demonstrate epidemiological outcomes within the sample.

## Results

### Descriptive statistics

Descriptions of the sample were categorised based on their demographics (Table 2) and reported symptomatology. 345 women were included in this study, but the information derived for each patient varied depending on the details completed in their notes.

**Table 2:** Demographic information of patients

Age at first surgery (removal)	(0,40]	(40,60]	(60,75]	(75,90]	Blank
	25	187	78	14	41
	7.25%	54.20%	22.61%	4.06%	11.88%
BMI	BMI < 30	BMI < 40	BMI > = 40	Unclear	Blank
	189	90	17	4	45
	54.8%	26.1%	4.9%	1.2%	13.0%
Ethnicity	Caucasian	Asian	African	Unclear	Blank
	310	9	4	8	14
	89.9%	2.6%	1.2%	2.3%	4.1%

#### Demographics

Based on the data, 54.2% and 22.61% of women have had their first mesh removal surgery between the ages of 40 and 60 and 60 to 75, respectively Approximately, 54.8% of patients have a BMI under 30, whilst the remaining are either obese or over the recommended BMI. Caucasian women dominate the sample with 89.9%. Within the sample Asians and Africans appear to be 2.6% and 1.2%, respectively. Due to the uneven distribution of ethnic groups within the sample, correlation of these to reported symptomatology data (Table 3) were limited. Variations in race is an important facet to further delineate any specific clinical outcomes to a particular group, although this was not feasible to the lack of data available.

**Table 3:** Patient reported symptoms

Para	0	1	2	3	>=4	Blank
	12	31	142	78	22	60
	3.5%	9.0%	41.2%	22.6%	6.4%	17.4%
CS	0	1	2	3	4	Blank
	267	18	3	0	1	56
	77.4%	5.2%	0.9%	0.0%	0.3%	16.2%
N years beginning of symptoms	0	[1,5]	[6,10]	[10,15]	>=16	Blank
	121	158	26	3	1	36
	35.1%	45.8%	7.5%	0.9%	0.3%	10.4%
N Years insertion-removal	W	[1,5]	[6,10]	[10,15]	>=16	Blank
	35	102	121	58	20	9
	10.1%	29.6%	35.1%	16.8%	5.8%	2.6%

Of the 273 women, all but 12 of them had been pregnant, with 9% having had one delivery, nearly 40% having had at least two deliveries and 28% having had 3 or more deliveries. 267 out of a total of 345 women reported their delivery status. 6.4% (23 women) had had at least one caesarean section, whereas 77.4% had a normal delivery (16.2% had no record of the mode of delivery).

#### Mesh types

**Fig. 1:**
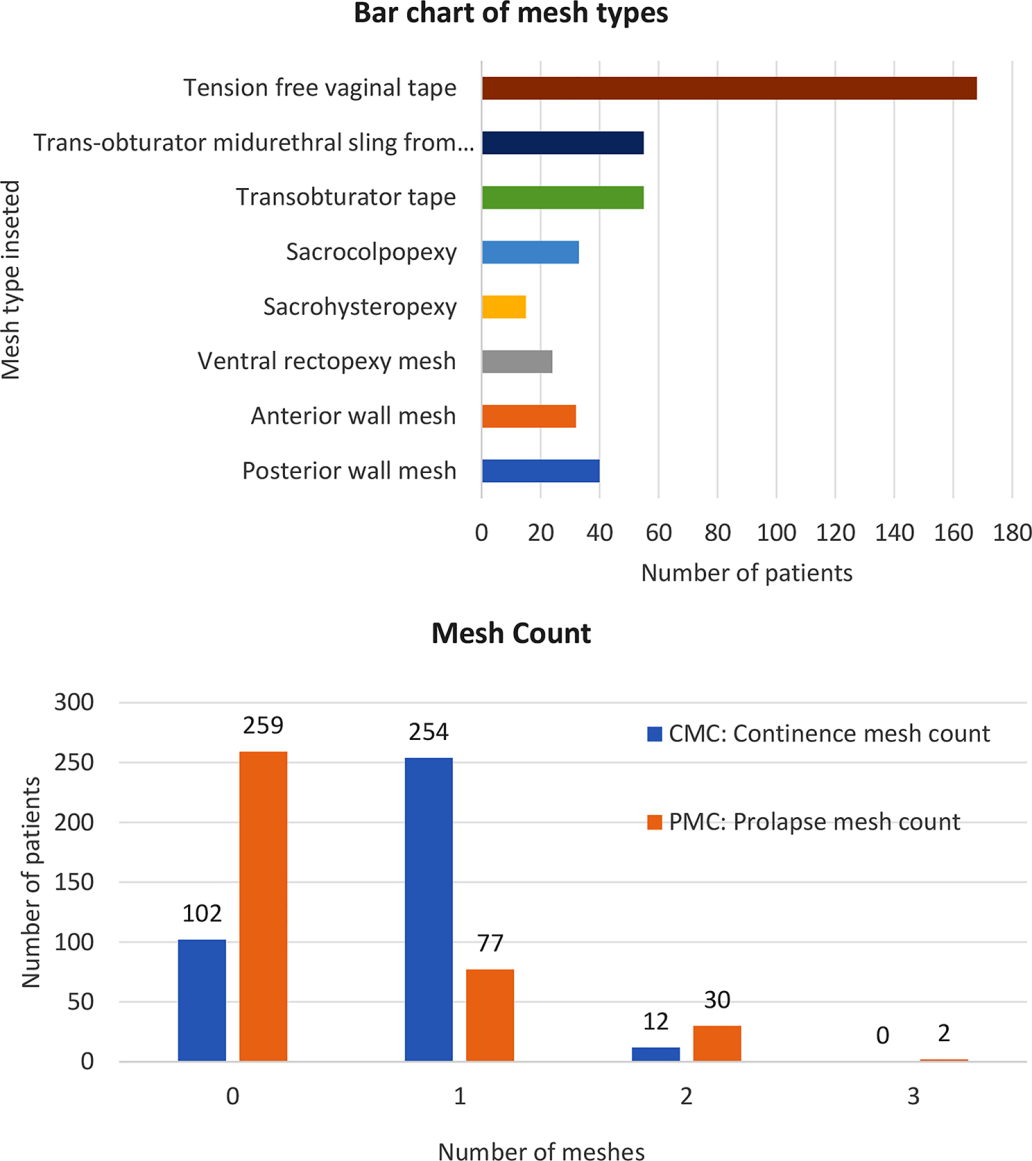
Bar chart of mesh types of patients and mesh counts

Most patient reported stress urinary incontinence and had a retropubic tension free vaginal tape/mesh. Obturator tapes/meshes formed the second largest group of mesh insertions, whereas other mesh types accounted for roughly 20% of those inserted, as demonstrated in Fig. 1.

**Fig. 2:**
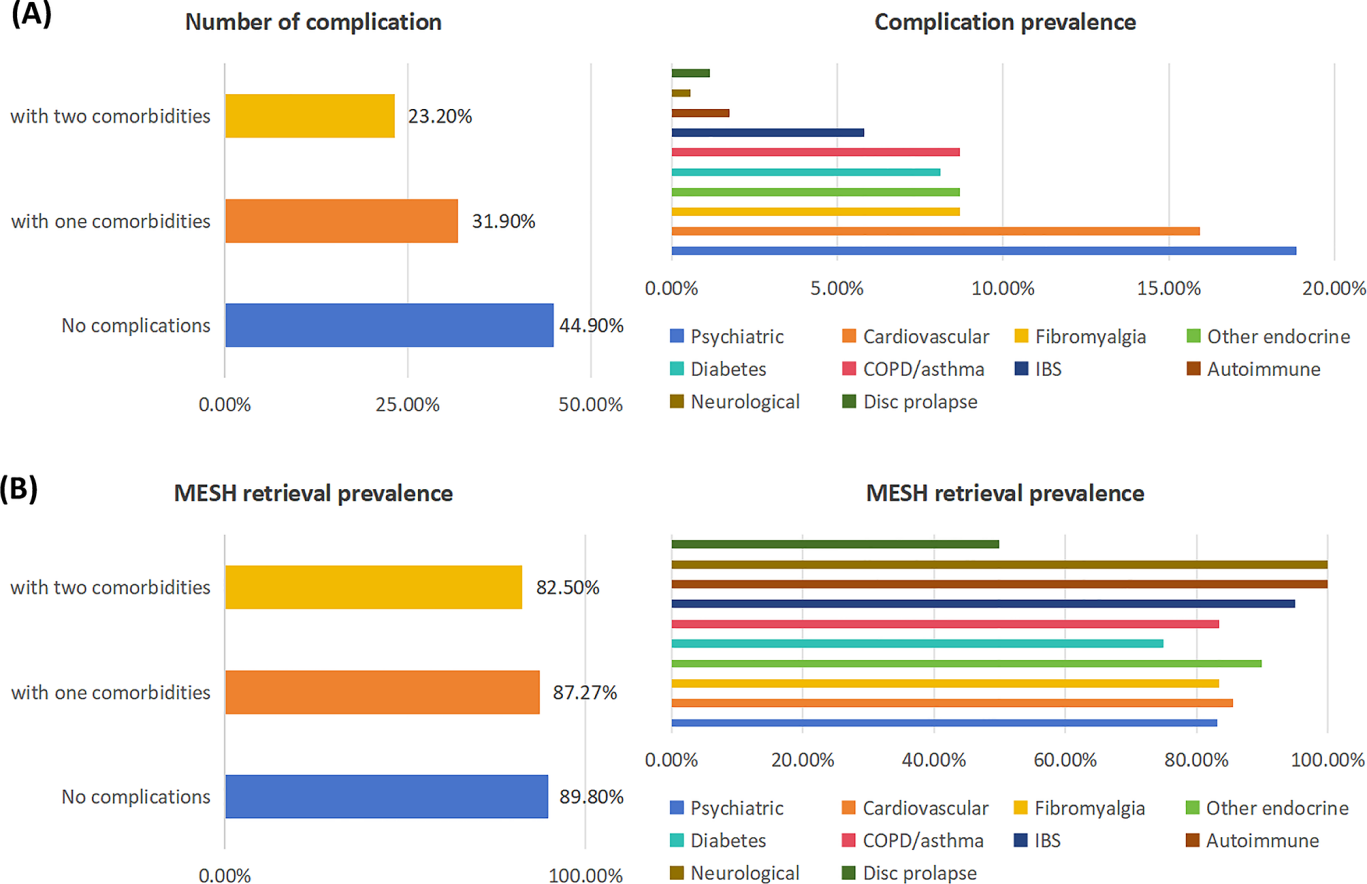
Bar chart of retrieval prevalence in patients with complications. (**A**) Prevalence of Patients with 0, 1, and 2 Comorbidities and Specific Comorbidity Breakdown” (**B**) Proportion of Patients with mesh retrieval among those with 0, 1, and 2 Comorbidities and Specific Comorbidity Types

Medical histories of 345 patients were unremarkable although complications varied from neurological to neuropsychiatric to gastrointestinal, as demonstrated in Table 4; Fig. 2.

**Table 4:** Information of medical history of patients

Complications (C)	History 1	History 2	Count	Prevalence (C)	Retrieval prevalence in patients with (C)
No complications	155	265	420	44.93%	89.8%
> 2 comorbidities	80	80	80	23.19%	82.5%
Psychiatric	43	22	65	18.84%	83.1%
Cardiovascular	42	13	55	15.94%	85.5%
Fibromyalgia	30	0	30	8.70%	83.4%
Other endocrine	20	10	30	8.70%	90%
Diabetes	19	9	28	8.12%	75.0%
COPD/asthma	15	15	30	8.70%	83.4%
IBS	12	8	20	5.80%	95%
Autoimmune	5	1	6	1.74%	100%
Neurological	2	0	2	0.58%	100%
Disc prolapse	2	2	4	1.16%	50%

Of the pooled patients, 84.6% did not report an autoimmune disorder whilst 91.9% reported other health conditions including osteoarthrosis (28, 8.1%) and rheumatic arthritis (2.0%). Chronic kidney disease (CKD), Sciatica and Ulcerative colitis, were reported by 0.9% patients along with diabetes. Cardiovascular disease and diabetes are common complications shared by patient with CKD and Sciatica, although this is limited in number within the sample size. In order to better evaluate a potential clinical correlation and overall disease burden, the sample size should be increased.

### Prevalence and incidence

To determine prevalence, we reviewed 2 specific composites from the sample, including the incidence of mesh insertion and removal, as well as the age at the first surgery and years till the removal to understand the overall impact of the surgical complication among the pooled patients. Therefore, the exposure within the group remained as mesh surgery due to incontinence. The prevalence for mesh removal was 89.85%. The incidence for mesh retrieval was 95.04% within the sample.

**Fig. 3:**
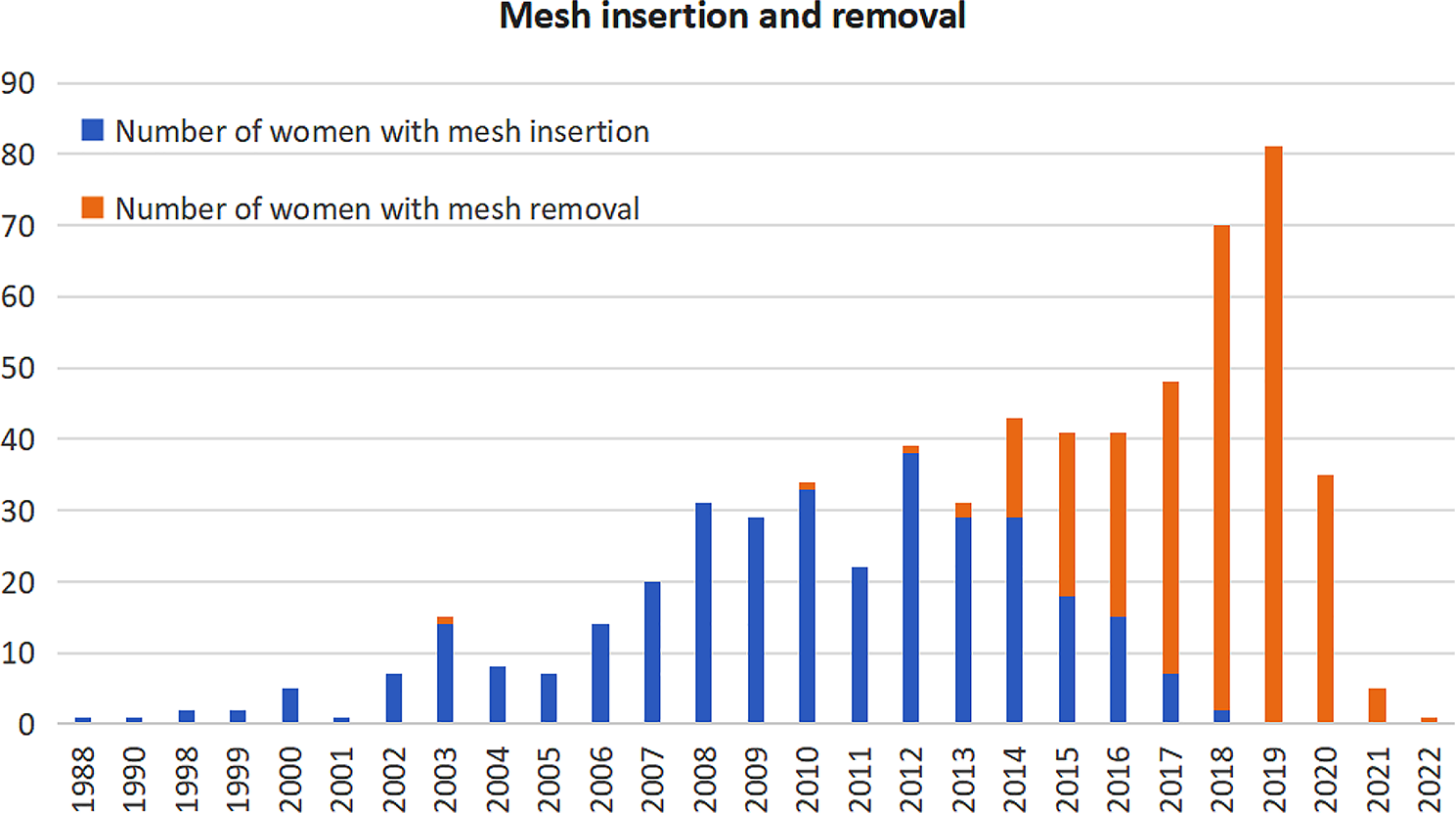
Histograms and line charts of mesh insertion and mesh removal

Figure 3 illustrates that most women had mesh insertion surgery between 2007 and 2014, whilst by 2015, most women were removing these within this cohort. The majority of removals were performed between 2018 and 2019.

**Fig. 4:**
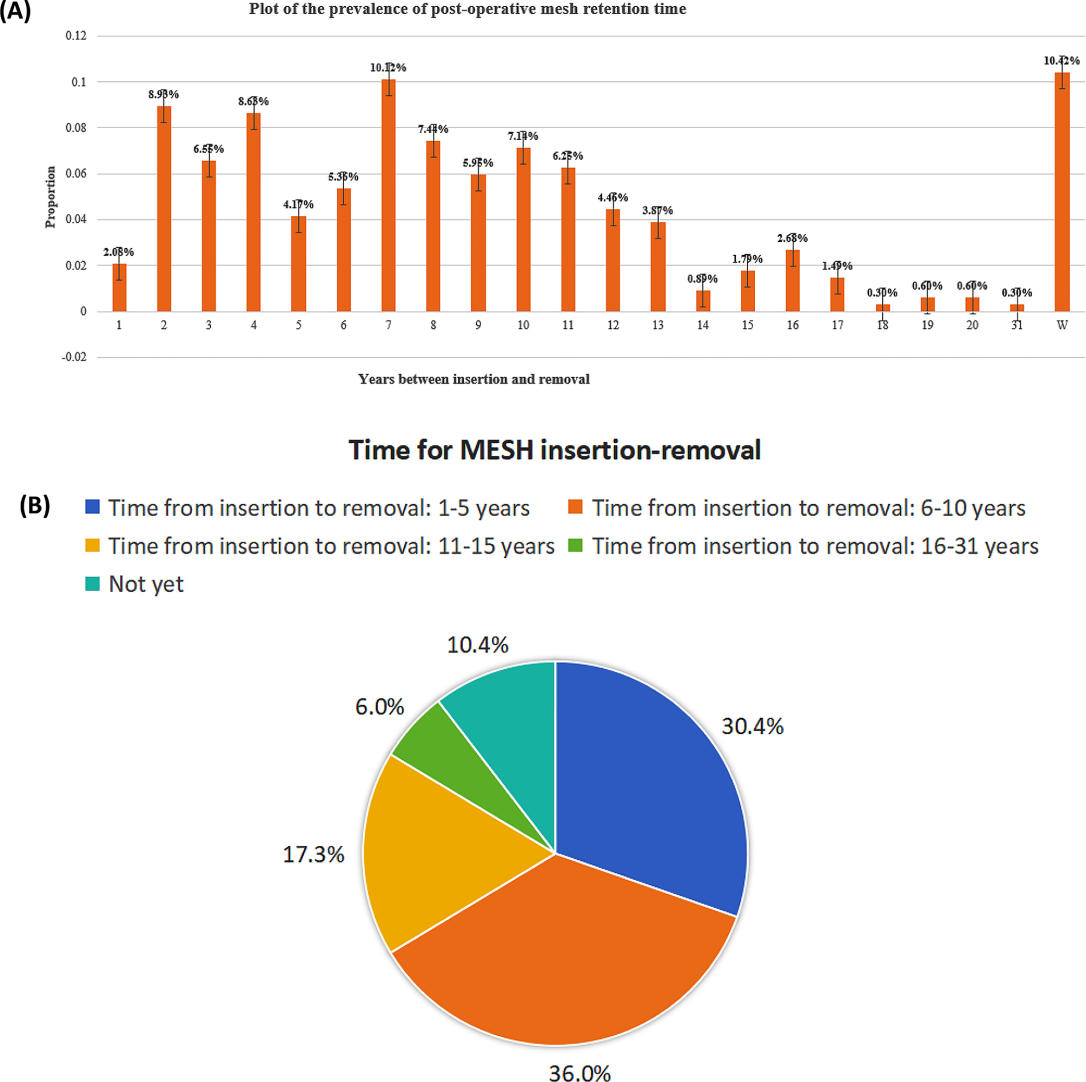
(**A**) Plot of the prevalence of POST-operative mesh retention time. (**B**) Pie chart of time for mesh insertion removal

The bar chart and pie chat (Fig. 4) show the proportions of people with mesh inserted based on the time intervals between insertion and removal.

### Data modelling

#### Correlation

**Fig. 5:**
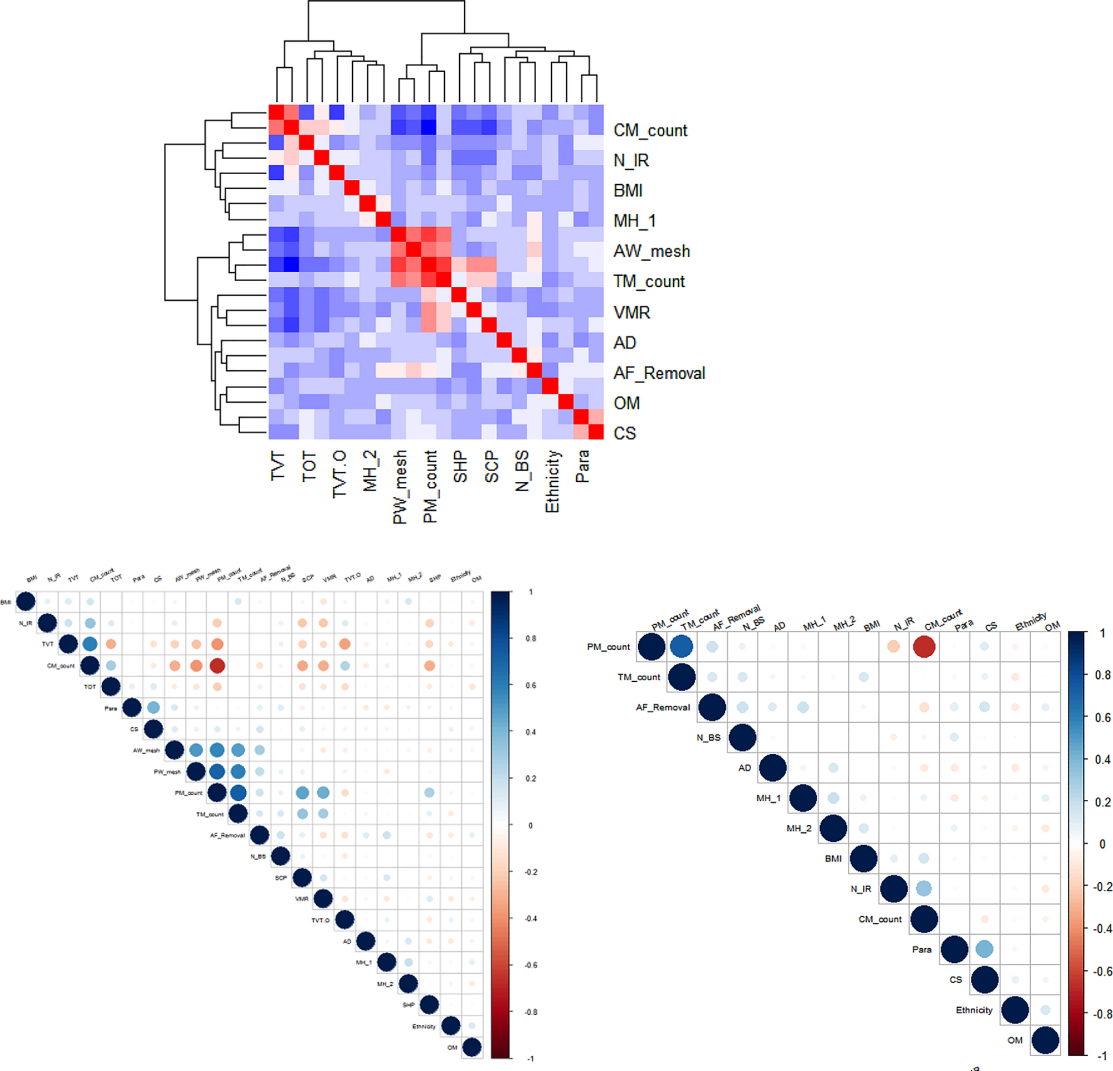
Correlations between outcome and variables

**Fig. 6:**
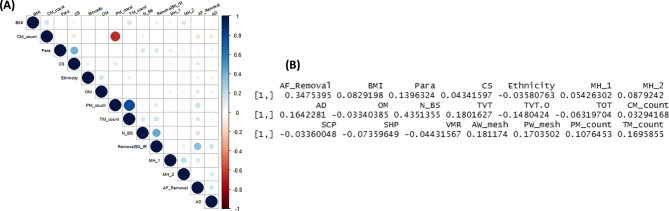
(**A**) Variable correlations for “Mesh Removal” group. (**BF**) Correlation between insertion-removal time intervals and other variables

To determine potential correlations between each of the patient reported symptoms having had mesh removal surgery, we reviewed underlying variables to deter a linear relationship mathematically as demonstrated in Fig. 5 and 6. The correlations between most variables are low, and the few variables that are correlated are due to numerical mathematical relationships between them.

The insertion to removal time show a mathematical correlation where the correlation coefficient is 0.348. Additionally, there appears to be a mathematical correlation of 0.435 between mesh insertion surgery and symptom reporting.

#### Group comparison

**Fig. 7:**
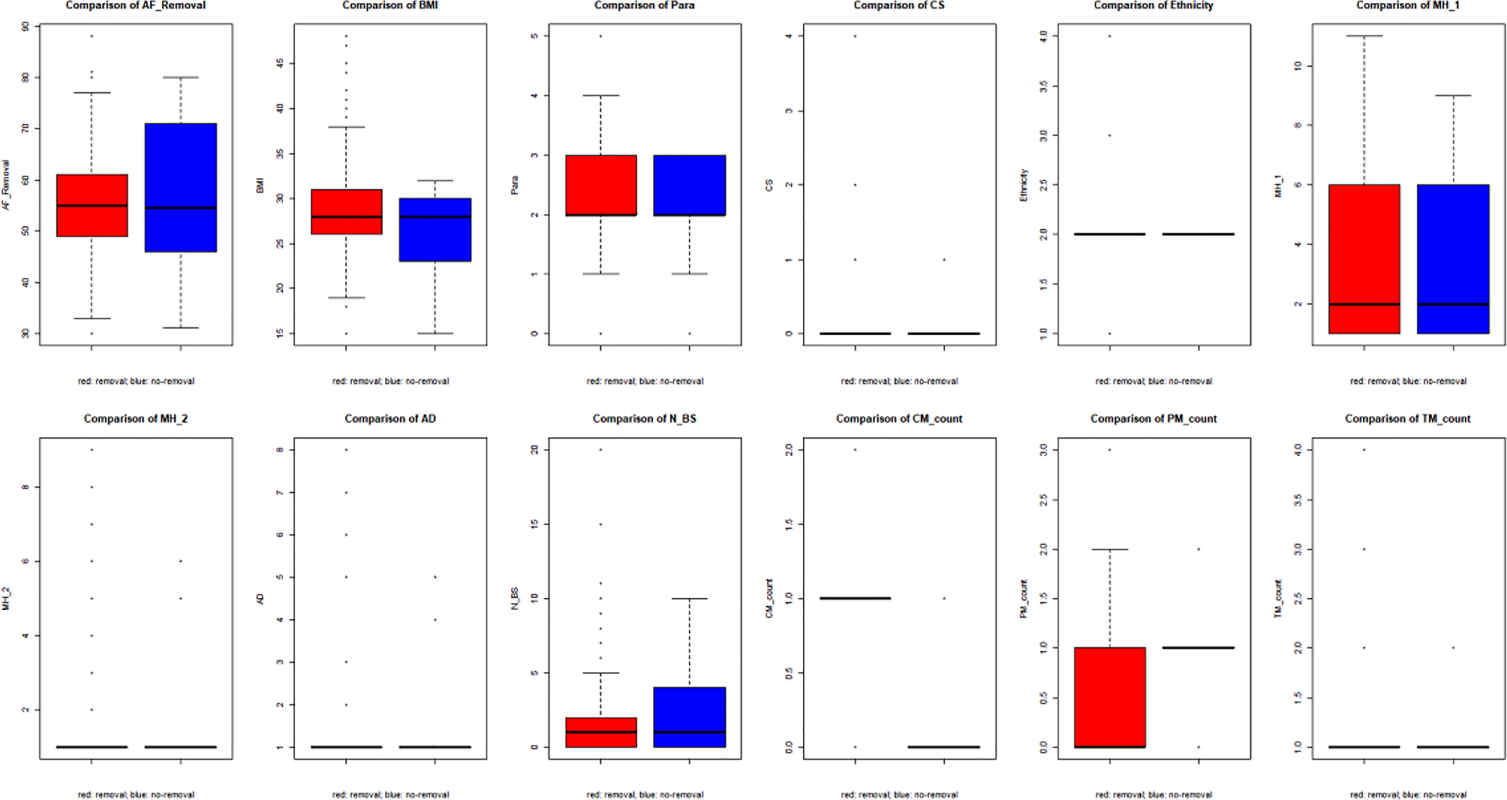
Comparison of variables among removal and non-removal groups

There appears to be no statistical significance difference between ethnicities and other symptoms in relation to mesh removal. This is apparent within Fig. 7.

## Discussion

The surgical management of SUI and POP from 1997 onwards was almost completely dependent on the use of mesh as a means to achieve functional and anatomical restoration of the pelvic floor [25–27]. Despite complications such as chronic pain, mesh erosion into pelvic organs and other problems [9, 15, 22, 28, 29] being well documented in the literature from 2000, the number of continence mesh insertions continued to increase in the UK exceeding 100,000 between the years 2008/2009 and 2016/2017, whereas prolapse mesh insertions exceeded 27,000 in the same time period. In 2016/2017 the insertion rate of mesh was reduced by 48% to 7245 annually, from 13,990 implants in 2008/2009. This was mirrored in prolapse mesh insertion, with a reduction of 13% to 2689 annually in 2016/2017, from 3073 in 2008/2009. However, this does not consider women with MAPS or mesh complications in primary care settings, who had not been referred to secondary or tertiary care for further management. The reduction in mesh insertion overall in those years were associated with mesh removal rates of between 7.3 and 10.2 patients per 1000 per year for continence mesh, and 1.8 and 3.9 per 1000 patients per year for prolapse mesh (https://digital.nhs.uk/news/2018/nhs-digital-publishes-statistics-on-vaginal-mesh-procedures). Mesh removal as defined within NHS digital coding records includes mesh that was trimmed, divided, partially or completely excised or stretched, unlike mesh that was removed in this cohort of patients where it was surgically removed specifically to reduce the mesh load significantly.

The demographics of the patients within our cohort requesting mesh removal surgery mirror that of women who were at risk of developing SUI or POP in the first place, necessitating continence and/or prolapse surgical management [30]. The majority of women who had continence mesh were Caucasian, had a BMI under 30, were middle-aged and over 96% had had at least one child. The mode of delivery was predominantly vaginal, which is another well recognized factor of developing SUI and POP [31–34]. Thus, these findings were not unexpected.

The onset of patient reported symptoms associated with the mesh, leading to women seeking surgical removal, predominantly commenced within the first 5 years of its insertion. However, well over a quarter of all patients developed symptoms after the mesh had been in situ for over six years. This suggests that complications can occur at any time from the mesh’s insertion – there is no time limit when it could be considered to be completely safe without it causing a potential adverse event. Perhaps the most worrying statistic is even though 80.9% of women had symptoms within the first five years of insertion of the mesh, the vast majority (64.7%) had to wait up to 10 years before the mesh was removed. The factors that may have influenced this are multi-factorial. Burki quoted from the *First Do No Harm* report by Baroness Cumberlege in 2020 [35] *“There is an institutional and professional resistance to changing practice even in the face of mounting safety concerns”*,* they wrote. “Mistakes are perpetuated through a culture of denial*,* a resistance to no-blame learning*,* and an absence of overall effective accountability.”* The average time from mesh insertion to removal was seven years. The Cumberlege report clearly explains why so many women had reasons for delayed access to care.

The commonest mesh inserted in almost 50% of this cohort of patients was the retropubic *TVT* (tension free vaginal tape) continence mesh, with just over 30% having an obturator continence mesh. The remaining patients had either an abdominal prolapse mesh (sacrohysteropexy, sacrocolpopexy or ventral mesh rectopexy); vaginal prolapse mesh (anterior or posterior vaginal wall mesh); or, a combination of the different mesh types. This is in keeping with statistics from NHS Digital which show that year on year the retropubic continence mesh was most commonly used mesh in women with SUI. Hence, it is not surprising that mesh removal surgery was predominantly within this group.

Although the medical histories of the 345 patients were unremarkable, the prevalence of cardiovascular disease higher than expected in women aged between 40 and 6o years of age, the largest number of women in this study. The prevalence of cardiovascular diseases within the UK population runs at 3–4% [36], where as in our group it exceeded 15%. Mental health issues conversely accounted for 18% of this cohort of patients, which is in keeping with expected national statistics of roughly 1 in 5 women having mental health problems (https://digital.nhs.uk/data-and-information/publications/statistical/adult-psychiatric-morbidity-survey/adult-psychiatric-morbidity-survey-survey-of-mental-health-and-wellbeing-england-2014) This suggests mental health issues were not the reason women were seeking mesh removal surgery, and the oft quoted reasons for clinicians not supporting the removal of mesh being because the patients symptoms were ‘*all in their heads’* does not stand.

Mesh insertion in the United kingdom commenced as early as 1997, and increased year on year until 2016/2017. It remains unknown just how many meshes were inserted in the UK, but based on the data published in 2018 by NHS digital it can be assumed that between 2003 and 2019 at least 10,000 meshes were inserted annually, for SUI and POP with a conservative estimated total of 160,000 implants in the UK. With a 9.8% hospital readmission for reparative surgery [37], this suggests that at least 15,680 women have or will have mesh complications necessitating medical and/or surgical management. In our study most insertion occurred between 2007 and 2014. By 2015 most women within our group were seeking removal surgery, with a significant amount of surgery being done between 2018 and 2019. With the cessation of mesh implantation, and the fact that women may develop complications as late as 15 years after implantation, the numbers are likely to rise in the next decade.

The correlation between each patient reported symptom and having had mesh removal surgery was low, suggesting though a relationship exists it is weak. Similarly, there was a low correlation between ethnicities and other symptoms in relation to mesh removal. However, there was a moderate correlation between mesh insertion surgery and symptom reporting, as well as between insertion and removal time. Given the heterogenous nature of this patient group and the complexity of their clinical presentation, it is not surprising that finding a data model that can help predict which patients will need to consider mesh removal surgery and to correlate that to their clinical features is challenging. There is growing interest in the use of observational data (or, ‘real-world data’) to assess the safety, effectiveness, and cost effectiveness of medical technologies, but less so in determining clinical parameters that can determine surgical intervention [38]. Furthermore, certain barriers may limit the use of data modelling in this patient group, including challenges in identifying and accessing relevant data, in ensuring the quality and representativeness of data, and in the differences between datasets in terms of their structure, content, and coding systems used. Coding for mesh removal surgery was only defined in 2012 by OPCS- - the statistical classification for clinical coding of hospital interventions and procedures undertaken by the NHS. It is mandatory for use by health care providers to support various forms of data collections for secondary uses, but for mesh removal surgery it did not exist for at least a decade after meshes were routinely used in clinical practice. This may explain the paucity of such data.

This retrospective evaluation of electronic healthcare data has shown that MAPS as a presenting factor was a significant component of women receiving mesh removal surgery. The rise in women accessing care from 2015 onwards, regardless of mesh inserted, may be due to the evolutionary history of mesh complications becoming more widely known and understood. Clearly, there has a been a progressive change in the narrative.

Continence and prolapse mesh insertion showed a perceptible decline from 2015 onwards. Several factors may have led to this including: the rulings by the FDA in 2011 banning mesh in the United States; the ruling by the MHRA in the UK in 2011 and 2012 raising awareness on the debilitating complications of the mesh; and the reports from the National Institute for Clinical Excellence between 2007 and 2017, where caution was urged in the use of mesh and a focus for more research was encouraged. But, perhaps it was the rise in public awareness led by women affected by the complications of mesh along with the national media that changed the emphasis from mesh insertion and its benefits, to that of mesh complications and the need for surgical removal [39, 40]. Unsurprisingly, this was mirrored in the rise of both medico-legal claims against implanting surgeons, device firms and institutions of practice [41]. The culmination in the Cumberlege report and the subsequent formation of the recommended nine national complex mesh centres, who’s focus is holistically managing women with continence and prolapse mesh complications, firmly establishes the place of mesh removal surgery in clinical practice.

With better data collection, the creation of data repositories and data modelling within the newly created complex mesh centres as of April 2021, more research in this newly created arena will help clinicians and patients better determine when surgery may be appropriate to help those with mesh complications. An extension of this study is clearly needed.

## Conclusions

All women who underwent mesh removal surgery presented to the clinic with a history of mesh associated pain syndrome and other comorbidities which may have influenced their decision. There were no specific predictors, other than chronic pain associated with mesh, determining which women underwent surgery, though those with continence mesh were more likely to do so. An association with mental health issues being a driver for pursuing surgery could not be demonstrated. However, it can be postulated a rise in public awareness of the problems women face after insertion of continence and/or prolapse mesh may have resulted in more women seeking surgery. In time, with better data repositories and prospective research, we will better understand the predictors for surgery in women with mesh complications.

## Data Availability

The data used in this retrospective evaluation of patients with mesh complication is derived from the records available within the electronic healthcare systems withn University College London Hospitals NHS Foundation Trust.
